# Insight into the effects of H_2_SO_4_ and HNO_3_ acidification processes on the properties of coal as an enhanced adsorbent for ciprofloxacin residuals: Steric and energetic studies

**DOI:** 10.3389/fchem.2023.1130682

**Published:** 2023-03-27

**Authors:** Ibrahim G. Al-Labadi, Marwa H. Shemy, Alaa Y. Ghidan, Ahmed A. Allam, Horváth M. Kálmán, Jamaan S. Ajarem, Jianmin Luo, Chuanyi Wang, Mostafa R. Abukhadra

**Affiliations:** ^1^ Department of Environmental Analysis and Technologies, Institute of Environmental Sciences, Hungarian University of Agriculture and Life Sciences, Gödöllő, Hungary; ^2^ Materials Technologies and Their Applications Lab, Geology Department, Faculty of Science, Beni-Suef University, Beni-Suef, Egypt; ^3^ Chemistry Department, Faculty of Science, Beni-Suef University, Beni-Suef, Egypt; ^4^ Department of Biological Sciences, Faculty of Sciences, The University of Jordan, Amman, Jordan; ^5^ Zoology Department, Faculty of Science, Beni-Suef University, Beni-Suef, Egypt; ^6^ Zoology Department, College of Science, King Saud University, Riyadh, Saudi Arabia; ^7^ School of Chemistry and Civil Engineering, Shaoguan University, Shaoguan, China; ^8^ School of Environmental Science and Engineering, Shaanxi University of Science and Technology, Xi’an, China; ^9^ Geology Department, Faculty of Science, Beni-Suef University, Beni-Suef, Egypt

**Keywords:** coal, acid treatment, ciprofloxacin, steric, energetic

## Abstract

A sub-bituminous natural coal sample (R.C) was treated with sulfuric acid (S.C) and nitric acid (N.C) as modified products and enhanced adsorbents for obtaining ciprofloxacin (CFX) antibiotic residuals from water. The characterization studied demonstrates enhancement in the surface area and the incorporation of new active oxygenated, sulfur-bearing, and nitrogen-bearing chemical groups into the structure of coal samples. This was reflected in the adsorption capacities that were enhanced from 164.08 mg/g (R.C) to 489.2 mg/g and 518.5 mg/g for N.C and S.C, respectively. The impact of the acid modification processes was evaluated based on the energetic and steric properties of their adsorption systems considering the parameters of the advanced monolayer equilibrium model with one energy site. The determined occupied active sites’ density of R.C (46.32–61.44 mg/g), N.C (168.7–364.9 mg/g), and S.C (159.2–249.9 mg/g) reflects an increase in the quantities of active centers after the acid treatment processes, especially with HNO_3_. The higher efficiencies of the active sites of S.C to adsorb more CFX molecules (*n* = 2.08–2.31) than N.C (*n* = 1.41–2.16) illustrate its higher adsorption capacity. The energetic investigation [adsorption (˂40 kJ/mol) and Gaussian (˂8 kJ/mol) energies] suggested adsorption of CFX by N.C and S.C mainly by physical processes such as van der Waals forces, hydrogen bonding, dipole bonding, and π–π interactions. Moreover, the determined thermodynamic functions including entropy, internal energy, and free enthalpy reflect the spontaneous and endothermic uptake of CFX on the surfaces of N.C and S.C.

## 1 Introduction

In the later decades, pollution of water sources with different species of organic pollutants was reported extensively as an associated result of the increase in chemical and pharmaceutical industries (Wang et al., 2020; Asadi-Ghalhari et al., 2022). Pharmaceutical residuals, petrochemicals, pesticides, dyes, surfactants, and drugs were reported extensively as essential organic pollutants that cause toxic and harmful effects on human, aquatic life, and wildlife (Mostafa et al., 2021; Abukhadra et al., 2022a). The discharged pharmaceutical residuals and their metabolite products attracted strong interest as agents causing hazardous environmental and health issues (Zhong et al., 2020; Fan et al., 2022; Tai et al., 2022). Commonly used antibiotics represent an essential category of the detected pharmaceutical residuals and organic pollutants in aqueous environments (Abukhadra et al., 2022a; Dhiman, 2022). Ciprofloxacin (CFX), levofloxacin, azithromycin, clarithromycin, and cefixime are the mostly detected antibiotic residuals in the aqueous environment and are associated with significant side effects according to the WHO (2017) (Rocha et al., 2017; Asadi-Ghalhari et al., 2022).

Ciprofloxacin (C_17_H_18_FN_3_O_3_) (CFX) is one of the fluoroquinolone analog antibiotics applied widely during the treatment of infectious diseases (bone, respiratory system, and gastrointestinal tract) and as an antibacterial agent against Gram-negative and Gram-positive bacilli (Abukhadra et al., 2022a; Falyouna et al., 2022; Guo et al., 2022). CFX exhibits poor metabolic stability, and about 75% of the delivered dosage in the animal or human body get rid of parent compounds (Abukhadra et al., 2020; Asadi-Ghalhari et al., 2022). Therefore, it is widely detected in the sewage systems of urban regions and hospitals at concentrations up to 150 μg/L and 30 mg/L in the discharged effluents of pharmaceutical factories (Peng Sun et al., 2009; Asadi-Ghalhari et al., 2022; Parmar and Srivastava, 2022). The existence of CFX in soil, water, and food chains is associated with remarkable health side effects such as kidney failure, fatty liver, vomiting, nausea, shivering, headache, and diarrhea (Zhang et al., 2021; Asadi-Ghalhari et al., 2022; Falyouna et al., 2022). Additionally, CFX residuals exhibit considerable catastrophic effects in supporting the resistance of common pathogens against the application of antibiotics (Xiang et al., 2020; Al-Musaw et al., 2021). Regarding its impact on the aquatic ecosystem, the CFX molecules cause perturbation in the nitrogen cycle as well as the translation and replication of chloroplasts in addition to its strong inhabitation impacts on the photosynthesis system, which negatively affects the growth rate of algae (Yin et al., 2017; Xiang et al., 2020; Falyouna et al., 2022). Therefore, the elimination of antibiotics as CFX effectively from the aqueous environments and the water supplies is a significant environmental challenge and a hot research topic.

Ozonation, advanced oxidation, adsorption, nano-filtration, membrane separation, ultrafiltration, ion exchange, flocculation, biological degradation, and coagulation are well-known remediation methods of organic compounds including pharmaceutical residuals (Grisales-Cifuentes et al., 2021; Abukhadra et al., 2022a). However, the adsorption elimination of the drug and pharmaceutical residuals was recommended to avoid the toxic properties of the resulting intermediate compound during oxidation and degradation (Boral et al., 2021; Grisales-Cifuentes et al., 2021; Salam et al., 2022). Moreover, the adsorption removal of pharmaceutical residuals is a simple, affordable, effective, safe, available, and reusable method at the industrial scale (Wang et al., 2020; Boral et al., 2021; Mostafa et al., 2021). However, there are several factors that control the selection of the appropriate absorbent, such as the production cost, fabrication procedures, availability of its precursors, adsorption capacity, reusability, kinetic rate, biodegradability, mechanical stability, surface reactivity, adsorption affinity, and environmental safety (Jiang et al., 2020; Abukhadra et al., 2022a). Therefore, innovative adsorbents based on natural raw materials that exhibit high availability and low cost were evaluated widely in the later periods, especially the carbonaceous or carbon-based structures (Giraldo et al., 2020; Ramirez et al., 2020; Boral et al., 2021; Grisales-Cifuentes et al., 2021).

Recently, most of the common ranks of natural coal were assessed as potentially low-cost and effective adsorbents of different species of synthetic dyes (Simate et al., 2016; Shaban et al., 2017; Surip et al., 2020). This demonstrates the activity of the coal chemical groups to act as adsorption centers for different organic molecules. As a mineralogical term, coal refers to an organic-rich sedimentary rock containing different macerals (cellulose, lignite, and resin) and inorganic impurities (Yu et al., 2018; Tang et al., 2019). As a chemical term, natural coal can be identified as having a series of aromatic polycyclic hydrocarbons in which the structural aromatic rings connect with different forms of oxygenated chemical groups (carbonyl, hydroxyl, phenolic, and carboxyl groups) that show significant adsorption activity (Kurková et al., 2004; Flores et al., 2019; Fonseca et al., 2020). Recently, some studies were carried out to enhance the surface activity and physicochemical properties of coal by different chemical and physical methods (Shaban et al., 2017; Ibrahim et al., 2021). This involved the chemical activation, thermal activation, demineralization, and metal oxide surface decoration of the coal structure (Simate et al., 2016; Shaban et al., 2017; Abukhadra et al., 2022a).

The previous studies reported remarkable enhancement effects of chemical or surficial modification processes on the properties of carbon-based materials. This includes activation of essential chemical groups, incorporation of new active chemical groups (oxygenated groups), and enhancement in the surface area (Jawad et al., 2018; Surip et al., 2020; Abukhadra et al., 2022b). Oxidation of coal by sulfuric acid was signified as a promising chemical activation method that enhances the electronegativity of its surface and induces the incorporation of additional active oxygenated groups into its structure (Jawad et al., 2018; Abukhadra et al., 2022b). Moreover, the modification of coal by sulfuric acid is a cost-effective process and can be performed by a single modification or activation step (Abukhadra et al., 2022b). Also, the previous studies demonstrated a significant effect of the modification of coal by nitric acid as low-cost oxidation reactions on the organic structure of coal by enhancing its hydrophilicity and providing the structure with numerous oxygen-containing functional groups (Shi et al., 2012; Elkady et al., 2020).

However, the efficiency of acid oxidation or modification of coal is controlled by the oxidation conditions, chemical activators used, and the concentrations of the used acid (Shi et al., 2012; Fonseca et al., 2020; Ibrahim et al., 2021). Unfortunately, the adsorption properties of acid-oxidized coal have not been assessed in satisfactory studies considering the types of the used acid and the species of organic pollutants. Therefore, this study involved, for the first time, the production of two acid-oxidized coal samples by sulfuric acid (S.C) and nitric acid (N.C) as enhanced low-cost adsorbents for CFX residuals from the aqueous environments. The adsorption properties were followed considering the main experimental factors in addition to detailed theoretical kinetic and equilibrium studies. Equilibrium behavior was illustrated based on the steric parameters (active site’s density, number of adsorbed CFX per site, and theoretical saturation adsorption capacities) and energetic parameters (adsorption energies, enthalpy, internal energy, and entropy) to assess the impact of the modification processes on the surface of coal as adsorbents in terms of the adsorbent/pollutant interface.

## 2 Methodology

### 2.1 Materials and methods

The coal used during the acid oxidation processes is sub-bituminous coal and was obtained from El-Maghara coal mine in Sinai, Egypt. The chemical composition of the used coal was detected based on the ultimate-proximate analyses and presented in Supplementary Table.S1. Sulfuric acid (H_2_SO_4_) (98% purity; cornel Lab Company) and nitric acid (HNO_3_) (65%; Sigma-Aldrich; Egypt) were applied during the oxidation and modification of the coal sample, respectively. CFX (C_17_H_18_FN_3_O_3_) fluoroquinolone antibiotic (≥98% (HPLC); Sigma-Aldrich; Egypt) was used as a source of antibiotic residuals during the adsorption experiments.

### 2.2 Synthesis of the oxidized coal adsorbents

First, the collected raw coal particles were ground by using a normal home blender to reduce the size of the coal fractions to determined range from 20 μm to 70 µm. After that, the obtained ground coal (10 g) was homogenized within two separate beakers of H_2_SO_4_ (95%; 100 mL) and HNO_3_ (65%; 50 mL) at a certain temperature (150°C) for 60 min. Then, samples modified by the two acids were washed extensively with distilled water to neutralize the surfaces of the oxidized products and remove the excess in the applied acids during the treatment processes. Finally, the products were dried for 10 h at 65°C using a digital dryer, kept in containers, and labeled as S.C (coal treated with sulfuric acid) and N.C (coal treated with nitric acid).

### 2.3 Characterization techniques

The impact of acid modification on the physicochemical properties of coal was assessed in terms of the changes in the structural, morphological, chemical, and textural properties. The structural properties of coal, S.C, and N.C were evaluated according to their XRD patterns using an X-ray diffractometer (PANalytical (Empyrean)) within the estimation range for the 2 Theta angle from 5^o^ to 80^o^. During the analysis, the diffraction data were recognized after exposing the samples as a powder to the X-ray radiation source at 1.5418 Å as the measuring wavelength (*λ*). The used X-ray is Cu–Kα–X-ray and the producing source is Cu anode, which is provided with 40 mA as the operation current and 40 kV as the determination voltage. The oxidation effects of the acids and the incorporated new chemical groups were identified based on the resulting Fourier Transform Infrared (FT-IR) spectra of the treated structures using an FT-IR spectrometer (FTIR−8400S) in the transmission mode within an estimation range from 4,000 to 400 cm^−1^. During the FT-IR investigation, the modified samples were milled with KBr powder at an adjusted ratio of 1:100, and then the mixtures were compressed by using a hydraulic press into pellets which were fixed in the sample holder of the FT-IR spectrometer. The morphologies of the acid-treated coal products in comparison with those of the raw sample were described according to their SEM images using a scanning electron microscope (Gemini, Zeiss-Ultra 55). Also, the impact of the treatment reactions on the surface area of the oxidized coal products was followed based on the recognized N2 nitrogen adsorption/desorption isotherm curves by using the Beckman Coulter surface area analyzer (SA3100 type) after degassing for 15 h at 105°C and the measuring temperature was adjusted at 77 K. The values of the specific surface area were calculated according to the Brunauer–Emmett–Teller (BET) equation, while the porous properties were calculated according to the Barrett–Joyner–Halenda (BJH) method.

### 2.4 Adsorption studies

The adsorption of CFX by S.C and N.S in comparison with raw coal was accomplished in the batch form considering the experimental effects of the essential parameters such as the pH (pH 2 to pH 9), adsorption duration (30 min to 1,080 min), and the CFX starting concentrations (100 mg/L to 800 mg/L) considering the change in the adsorption temperature from 298 K to 318 K. Moreover, the coal dosages and the tested CFX volumes were assessed at fixed values of 0.2 g/L and 250 mL, respectively. All these adsorption experiments were repeated for three runs, and the obtained average results were used for plotting of the results. The remaining CFX concentrations after conducting the desorption experiments were measured by the HPLC system (Merck/Hitachi) after the removal of coal particles. The applied HPLC system during the determination processes consists of a Luna column (150 mm × 4.6 mm), a pump (L-7100), and a detector (L-7400) in addition to the injection valve (Rheodyne 7725i), which is attached to the sampling loop (20 mL). The column (Phenomenex, Torrance, USA) of the used HPLC system consisted a stationary phase composed of PFP with 5 mm thickness. During CFX determination, a prepared mixture of distilled water and analytical grade methanol (60/40 volume ratio) was used as the mobile phase and introduced to the systems at an operative flow rate of 1 mL/min and 10 µL as an injection volume.

The measured remaining CFX concentrations were applied during the calculation of the actual or the experimental CFX adsorption capacities (Q_e_) according to Eq. 1, where C_o_, C_e_, V, and m are the starting CFX concentration (mg/L), remaining CFX concentration (mg/L), tested volume (mL), and the dosage of coal dosage (mg), respectively.

$$Q_{e\;\left(mg/g\right)}=\frac{\left(C_{o}-C_{e}\right)V}{m}\;.$$ (1)

The kinetic and traditional properties of the CFX adsorption processes by S.C, N.C, and raw coal were followed based on the non-linear fitting degrees with descriptive models (Supplementary Table.S1) involving the values of the obtained determination coefficient (*R* ^2^) (Eq. 2) and Chi-squared (χ^2^) (Eq. 3). Regarding the advanced isotherm studies based on the suggested models according to the theory of statistical physics (Supplementary Table.S1), non-linear fitting degrees were assessed considering the determination coefficient (*R* ^2^) and the recognized root mean square error (RMSE) (Eq. 4). The m′, *p*, Qi _cal_, and Qi _exp_ in the equation denote the inserted adsorption data, evaluated experimental variables, theoretically obtained CFX uptake capacity, and experimentally detected CFX uptake capacity, respectively.

$$R^{2}=1-\frac{\sum {\left(Q_{e,\exp}-Q_{e,cal}\right)}^{2}}{\sum {\left(Q_{e,\exp}-Q_{e,mean}\right)}^{2,}}$$ (2)

$$\chi^{2}=\sum \frac{{\left(Q_{e,\exp}-Q_{e,cal}\right)}^{2}}{Q_{e,cal}},$$ (3)

$$RMSE=\sqrt{\frac{\overset{m}{\underset{i=1}{\sum }}{\left({Qi}_{cal}-{Qi}_{\exp}\right)}^{2}}{m^{\prime }-p}.}$$ (4)

## 3 Results and discussion

### 3.1 Characterization of the adsorbent

**FIGURE 1:**
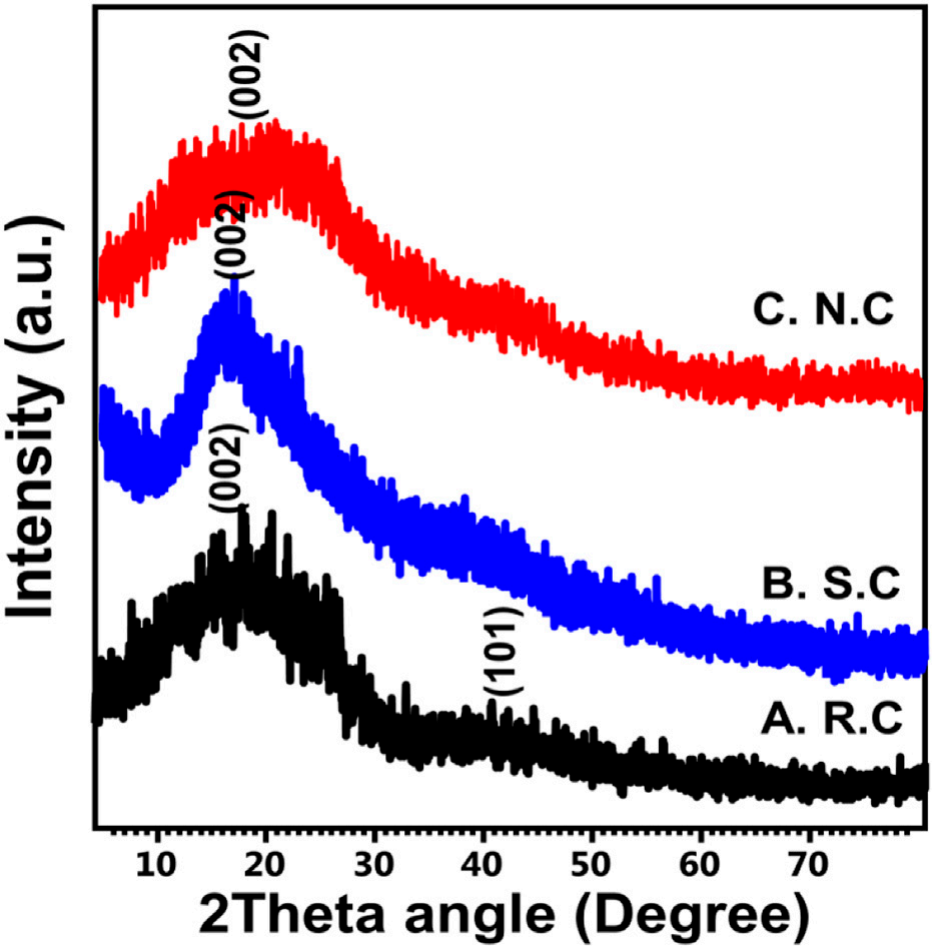
XRD patterns of raw coal (R.C) **(A)**, coal treated with sulfuric acid (S.C) **(B)**, and coal treated with nitric acid (N.C) **(C)**.

The structural properties of the S.C and N.C products were compared with those of the original coal in terms of the obtained XRD pattern (Figure 1). The raw sample shows the common diffraction pattern of amorphous aromatic and randomly oriented carbon with its recognizable broad peaks (8^o^–30^o^ (002) and 40^o^–50^o^ (101), respectively (Figure 1A) (Akinfalabi et al., 2017; Wong et al., 2020). This signifies the amorphous nature of the raw coal sample used as a precursor during the oxidation modifications. After oxidizing the sample with H_2_SO_4_ (S.C), the resulting pattern reflected deviation in the first peak (10^o^–32^o^) and significant reduction in the second peak (Figure 1B). This suggests significant destruction of the C-O-C bonds, which, in turn, increases the amorphization properties during the dehydration of the treated coal into polyaromatic/carbon (Niu et al., 2018; Ibrahim et al., 2021). Similar observations were detected in the XRD pattern of N.C ( coal modified by nitric acid); the main peaks deviated and reduced slightly as compared with the original sample but not to a significant degree as detected in the pattern of S.C (Figure 1C). This suggested high dehydration and oxidation effect of sulfuric acid on the structure of coal as compared to nitric acid.

**FIGURE 2:**
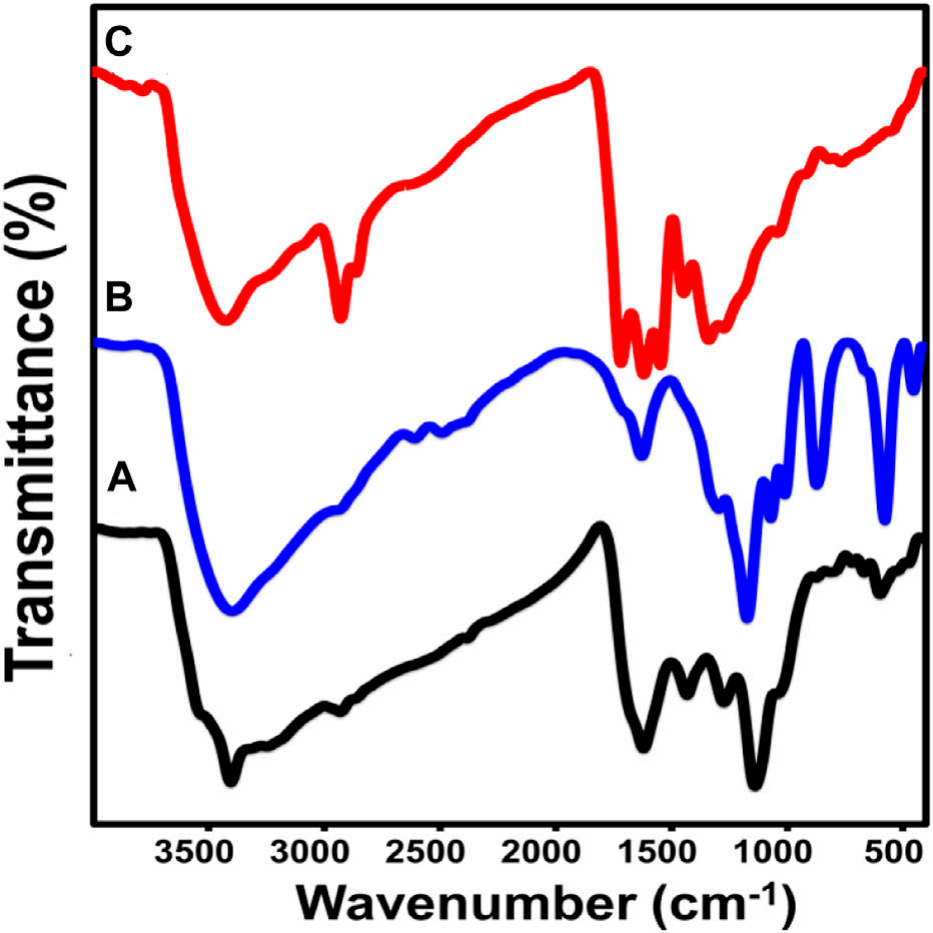
FT-IR spectra of raw coal (R.C) **(A)**, coal treated with sulfuric acid (S.C) **(B)**, and coal treated with nitric acid (N.C) **(C)**.

The FT-IR spectra of S.C and N.S, in comparison with the raw sample, also reflect the chemical and structural effect of acid treatment (Figure 2). The spectrum of the used raw sample shows the well-known bands of the coal chemical structure such as aromatic C-H (500–900 cm^*−*1^); aliphatic CH, CH_2_, and CH_3_ (2,858–2,940 cm^*−*1^); C-H in the methyl group (1,372 cm^−1^); C-H in the methylene group (1,450 cm^−1^); C-O group (1,000–1,200 cm^−1^); C=C (1,616 cm^−1^); -C=O (1716 cm^-1^); and hydroxyl groups (3,000–3,600 cm^−1^) (Figure 2A) (Shaban et al., 2017; Yu et al., 2018; Boral et al., 2021). Regarding the FT-IR spectrum of the coal treated by sulfuric acid, remarkable deviation and intensification of the OH identification band (3,398 cm^−1^) and reorganization of the new band related to the carboxylic groups (C=O) (1712 cm^−1^) (Figure 2B) are observed. These observations validate the significant oxidation effect of sulfuric acid on the structure of coal and the incorporation of additional oxygenated active chemical groups (–COOH) (Wong et al., 2020; Mateo et al., 2021). Moreover, there are several new bands reflecting the incorporation of several sulfur-bearing chemical groups, such as ^−^SO_3_H (1,133 cm^−1^), symmetric O=S=O (1,025 cm^−1^), asymmetric O=S=O (1,001 cm^−1^), and C-S (578 cm^−1^) (Figure 2B) (Fonseca et al., 2020; Mateo et al., 2021). The obtained spectrum of N.C (coal treated by nitric acid) also shows a considerable deviation in the position of the essential bands of raw coal, especially the OH group (3,355 cm^−1^). Additionally, there is a significant reorganization for the new band of the C=O stretching (1,716.16 cm^−1^) and the incorporated nitrogen-bearing groups (N=O stretching and C-N stretching (1,545.9 cm^−1^) (Figure 2C) (Alvarez et al., 2003; Shaban et al., 2017). This also confirms the oxidation effect of nitric acid on the coal structure and the incorporation of new active nitrogen-bearing chemical groups.

The FT-IR results were supported by the determined values of acid density and the elemental composition of the modified samples in comparison with the raw sample (Table 1). The acid density of S.C and N.C samples is 8.4 and 5.33 mmol/g, respectively, demonstrating the higher effect of H_2_SO_4_ than that of HNO_3_ (Table 1). Also, the remarkable increase in the S and N content of the treated products as compared to the raw coal confirm the oxidation effect of the acids used and the incorporation of new sulfur and nitrogen chemical groups in the S.C and N.C products, respectively (Table 1).

**TABLE 1:** Chemical composition, acid density, and surface area of R.C, S.C, and N.C samples.

	R.C	N.C	S.C
C (Wt., %)	68.4	53.2	48.8
S (Wt., %)	2.32	2.12	7.6
N (Wt., %)	2.53	9.3	7.6
H (Wt., %)	6.3	8.72	10.8
O (Wt., %)	10.6	19.7	24.4
Acid density (mmol/g)	0.064	4.62	8.4
Surface area (m^2^/g)	5.4	18.3	26.4
Pore volume (cm^3^/g)	0.011	0.41	0.48
Pore size distribution (nm)	2–100	2–86	2–80
Average pore diameter (nm)	50.2	34.8	22.4

The previous increment in the sulfur content was assigned mainly to the incorporated sulfur-bearing chemical groups during the modification of the coal with sulfuric acid or what is known as sulfonation reactions. The sulfuric acid causes significant adsorption for the hydrogen ion of the coal structure by hydroxyl groups, which are attributed to the entrapped acid molecules. This is accompanied by strong destructive effects on the present oxygen–hydrogen chemical bonds in addition to significant interaction between the protonated oxygen and lone pair electrons (Tang et al., 2019). Such interactions produce new types of Pi bonds with the entrapped or the adsorbed sulfur ions, forming protonated sulfur trioxide, which acts as effective electrophiles in addition to the sulfur ions (Yu et al., 2018). The chemical attack of the benzene rings within the coal structure with the sulfur ions results in the strong destruction of the double bonds of these aromatic rings, which induces the entrapping of new sulfur-bearing functional chemicals. Also, the chemical reactions between the coal structures and the sulfuric acid resulted in production of numerous HSO_4_^−^ radicals as very effective base radicals during the removal of hydrogen protons (Ibrahim et al., 2021). The incorporation of nitrogen is related to the impact of nitration on the present aromatic rings. Nitration involved electrophilic substitution where NO_2_^+^ ions are incorporated within the aromatic rings, forming *σ* combination. This results in new forms of nitrobenzene compounds, causing an increase in the oxygen and nitrogen content in the treated sample (Shi et al., 2012).

**FIGURE 3:**
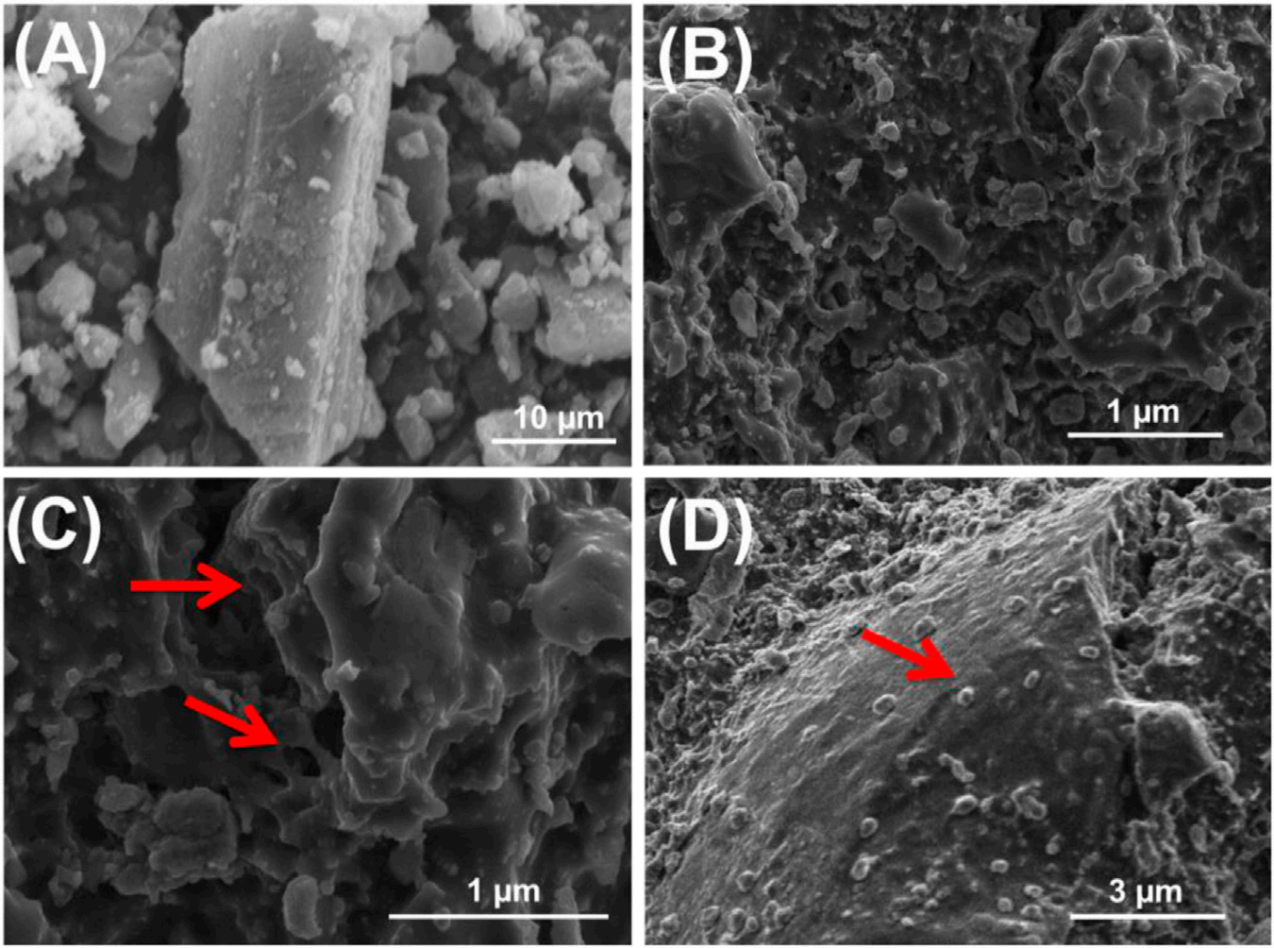
SEM images of raw coal (R.C) **(A)**, coal treated with sulfuric acid (S.C) **(B,C)**, and coal treated with nitric acid (N.C) **(D)**.

Regarding the morphological effect of the acid modification process on the coal, the obtained SEM images reflected considerable changes in the surficial features (Figure 3). The coal particles were observed in their common forms as compacted layers of massive or irregular shapes related to the compaction of their components of wood tissues and macerals (Figure 3A). The particles treated by H_2_SO_4_ exhibit rugged surfaces of irregular topography in addition to numerous exposed nano-nudes and few micropores and/or vugs, which might be related to the leaching effect of the acid on the present impurities (Figures 3B, C). The sample treated by HNO_3_ exhibits no observable changes as compared to the previously reported oxidation effects on the coal compacted layers, except for the decorated nano-nudes on their surfaces, which can considerably affect the textural properties of the modified sample (Figure 3C). There is a remarkable increment in the total pore volume from 0.011 cm^3^/g for raw coal to 0.41 cm^3^/g and 0.48 cm^3^/g for N.C and S.C products, respectively. Moreover, this is associated with a decrease in the pore size distribution properties (2–100 nm (raw coal), 2–86 nm (N.C), and 2–80 nm (S.C)) and the estimated average pore diameters (50.2 nm (raw coal), 34.8 nm (N.C), and 22.4 nm (S.C)). The increase in the pore volume is related to the significant increment in the micropores as a result of the dissolving and leaching of entrapped metal and carbonate impurities. Additionally, the changes in the topography of the coal particles strongly affected the determined values of pore diameters and pore volumes. Such enhancement in the pore volumes and formation of micropores results in a significant enhancement in the surface area of coal (5.4 m^2^/g), which increased to 26.4 m^2^/g and 18.3 m^2^/g after its structural modification with H_2_SO_4_ and HNO_3_, respectively, which can strongly affect the adsorption properties (Table 1).

### 3.2 Adsorption of CFX molecules

#### 3.2.1 Effect of pH

**FIGURE 4:**
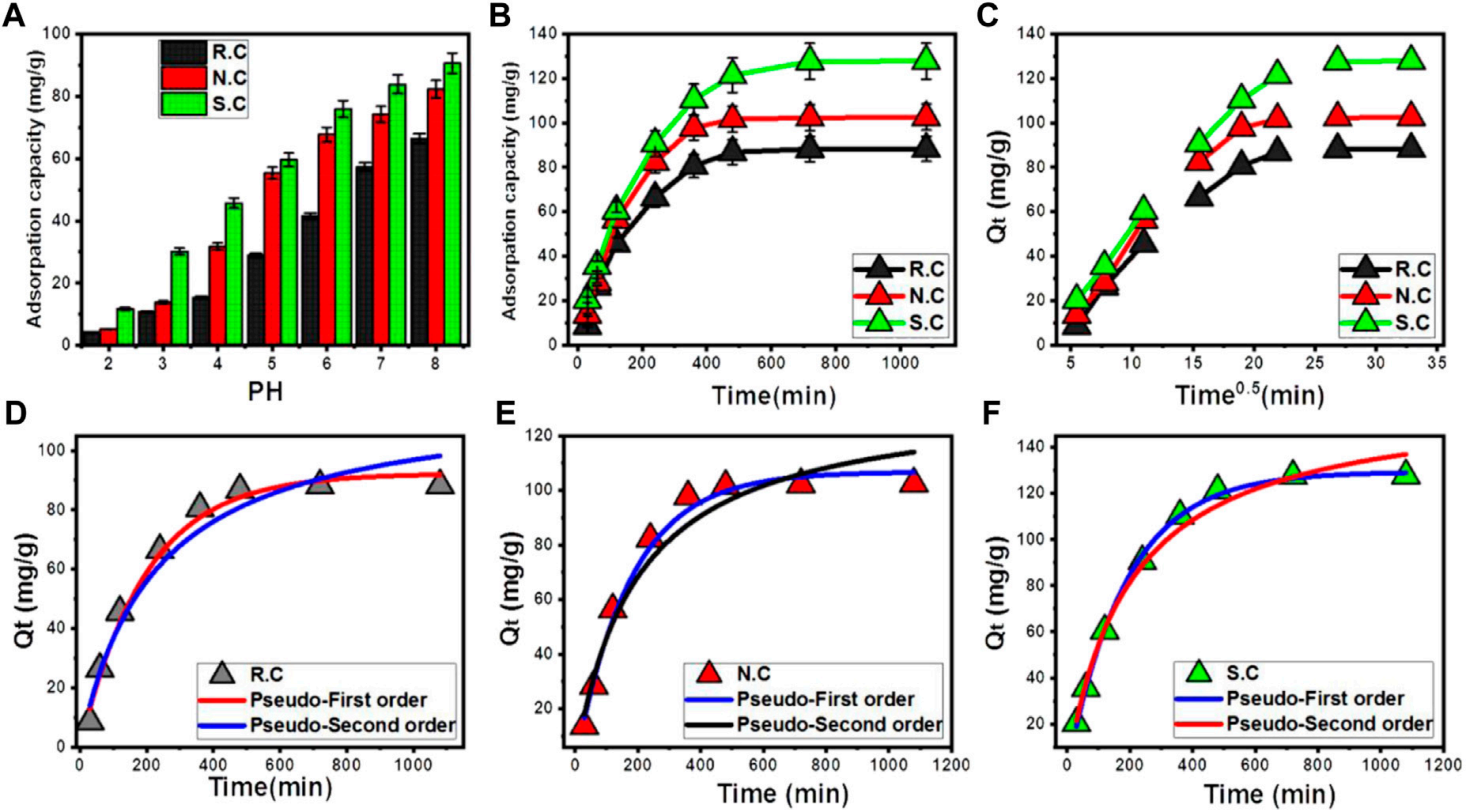
Experimental influence of the solutions’ pH on the adsorption of CFX **(A)**, effect of the adsorption duration on the adsorption of CFX **(B)**, the intra-particle diffusion curves of the CFX uptake results **(C)**, fitting of the CFX uptake results by R.C with the kinetic models **(D)**, fitting of the CFX uptake results by N.C with the kinetic models **(E)**, and fitting of the CFX uptake results by S.C with the kinetic models **(F)**.

The solution pH strongly affected the adsorption affinities of the studied adsorbents toward the dissolved ions either by controlling the ionization behaviors of the dissolved ions or by controlling the surficial charges of the used solid adsorbents. The changes in the CFX adsorption properties of raw coal (R.C), S.C, and N.C with the adjusted pH of the solutions were addressed from pH 2 to pH 8. The essential experimental factors were considered at fixed values (0.2 g/L (coal dosage), 240 min (adsorption duration), 200 mg/L (CFX concentration), 250 mL (volume), and 298 K (adsorption temperature)). The observed CFX uptake results validate the enhancement with adjustable increments in the pH of the solutions until pH 8 (66.4 mg/g (R.C), 82.3 mg/g (N.C), and 90.6 mg/g (S.C)) (Figure 4A). At pH 9, the R.C samples and the modified products of N.C and S.C show a decline in the achieved CFX uptake properties. This behavior is affected mainly by the zwitterionic nature of CFX antibiotics and the surface charges of the used coal adsorbents. The speciation properties of CFX demonstrate two pKa values [pKa1 = 6.1 and pKa2 = 8.7 (pKa2)] (Gor and Dave, 2020; Abukhadra et al., 2022c). Therefore, the dissolved CFX molecules are of cationic form at pH values lower than 6.1, anionic form at pH higher than pKa2, and zwitterionic form within the pH range from 6.1 to 8.7 (Najafpoor et al., 2019). Positive hydronium ions on the surfaces of R.C, N.C, and S.C at low pH conditions (˂ pH 6) resulted in significant electrostatic repulsion with the cationic phases of CFX. The high alkaline conditions (pH > 8) induce saturation of the coal adsorbents with the negative charges of the hydroxyl ions, which also exhibit repulsion with the present anionic phases of CFX. Therefore, the pH range from 6–8 preserves the sufficient positive charges to adsorb the formed zwitterionic and anionic phases of CFX (Najafpoor et al., 2019). This was supported by the determined pH_(ZPC)_ values of R.C (6.6), N.C (6.2), and S.C (5.4), which demonstrate the high saturation of their surfaces with negative charges beyond these values.

#### 3.2.2 Kinetic studies

##### 3.2.2.1 Time interval

The changes in the CFX adsorption properties of R.C, N.C, and S.C by increasing the uptake duration were followed from 30 min to 180 min. The essential experimental factors were considered at fixed values (0.2 g/L (coal dosage), pH 8 (adsorption pH), 200 mg/L (CFX concentration), 250 mL (volume), and 298 K (adsorption temperature)). There are significant variations in the actual CFX adsorption rates by R.C, N.C, and S.C, with increasing the adsorption duration as observed evidently in the segmental forms of the presented curves (Figure 4B). First, the first parts of the uptake curves display steep slopes validating abrupt adsorption of CFX causing strong variation in the quantities of the adsorbed antibiotic molecules. This can be recognized until 480 min, and after this interval, the applied R.C, N.C, and S.C exhibit low adsorption rates and neglected variation or nearly fixed adsorbed quantities of CFX until the end of the assessed duration interval (1,080 min) (Figure 4B). This denotes the equilibrium states of R.C, N.C, and S.C as the applied adsorbents of CFX achieving 88.3 mg/g, 102.5 mg/g, and 127.8 mg/g equilibrium capacities, respectively (Figure 4B). The high availability of the free, effective, and active sites or receptors throughout the surfaces of R.C, N.C, and S.C during the initiation of the processes induces the abrupt adsorption of CFX. The increment in the CFX adsorption duration is associated with the decline in the available free sites as a result of their continuous consumption by the adsorbed antibiotic molecules. After the occupation of all these sites with the CFX molecules, the used R.C, N.C, and S.C attain their saturation states, which are known as the equilibrium states, and they cannot adsorb CFX molecules anymore (El-Sherbeeny et al., 2021).

##### 3.2.2.2 Intra-particle diffusion behavior

The intra-particle diffusion properties of R.C, N.C, and S.C as the used adsorbents of CFX reflected segmental behaviors (Figure 4C). This is normally associated with changes in the dominant mechanisms during the increase of the adsorption duration of CFX in addition to the predicted impact of molecule diffusion toward the effective centers on the surfaces of R.C, N.C, and S.C (El Qada, 2020; Salam et al., 2020). The change in the CFX uptake mechanisms might include A) external surface (border) uptake processes, B) intra-particle diffusion processes, and C) equilibrium and saturation stages (Lin et al., 2021). The initial CFX adsorption processes based on the first marked segment signify the external or surficial uptake of the antibiotic molecules, and this process is regulated essentially by the availability of the effective receptors of R.C, N.C, and S.C (Figure 4C) (Albukhari et al., 2021). By observing the second segment, which appears along the intermediate portions or parts of the curves, the impact of the CFX surficial uptake declined or diminished significantly. Moreover, new processes were identified as the controlling mechanisms related to the intra-particle diffusion effects and layered uptake reactions (Lin et al., 2021; Abukhadra et al., 2022a). By the end of CFX duration, the third segment was identified, which involved the equilibrium states of R.C, N.C, and S.C (Figure 4C). This segment connotes the complete consumption or occupation of all the effective binding sites and remarkable formation of thick layers of adsorbed CFX antibiotics on the R.C, N.C, and S.C structures (Salam et al., 2020; Sayed et al., 2022). Additionally, the adsorbed CFX during this stage is controlled by different types of molecular interaction and/or the interionic attraction processes (Jiang et al., 2020).

##### 3.2.2.3 Kinetic modeling

The CFX adsorption kinetics in the presence of R.C (Figure 4D), N.C (Figure 4E), and S.C (Figure 4F) as solid adsorbents was described according to the common theoretical hypothesis of pseudo-first-order (PFO) and pseudo-second-order (PSO) models. The values of determination coefficient (*R* ^2^) and Chi-squared (χ^2^) were presented as the controlling factors of the fitting degrees of the non-linear fitting processes with the two assessed kinetic models (Table 2). Fitting of the CFX uptake results by R.C, N.C, and S.C with the non-linear equation of the PFO model reveals lower X^2^ and higher *R* ^2^ values than the obtained values for the PSO model (Table 2). Therefore, the occurred reaction during the CFX uptake processes by R.C, as well as its acidified or oxidized products, follows predominantly the kinetic behaviors of the PFO kinetics and is affected strongly by the impacts of physisorption, such as the electrostatic attractions (Huang et al., 2018; Sherlala et al., 2019). The previous fitting results were also supported by the considerable similarity between the obtained Q_e_ values by experimental detection (88.3 mg/g (R.C), 102.5 mg/g (N.C), and 127.8 mg/g (S.C)) and theoretically obtained values as a parameter of the PFO model (92.25 mg/g (R.C), 106.7 mg/g (N.C), and 129.3 mg/g (S.C)) (Table 2). However, the observable high fitting of the CFX uptake results using R.C, N.C, and S.C with the PSO kinetic model validates considerable impacts of some weak chemisorption processes (electron exchange, internal diffusion, surface complexation, and electron sharing) as associated mechanisms during the occurrence of the dominant physisorption processes (Sherlala et al., 2019; Salam et al., 2020). Both physisorption and chemisorption reactions might occur on the surfaces of R.C, N.C, and S.C at the same time. This might be result in the formation of a layer of the physically adsorbed CFX molecules above another layer of chemically adsorbed CFX molecules by R.C and its oxidized samples of N.C and S.C (Jasper et al., 2020).

**TABLE 2:** Mathematical parameters of the studied kinetic models.

Kinetic models			
	**Model**	**Parameters**	**Values**
R.C	Pseudo-first-order	K_1_ (1/min)	0.005
		Q_e__(Cal)_ (mg/g)	92.25
		R^2^	0.98
		X^2^	0.50
	Pseudo-second-order	k_2_ (mg/g min)	3.77 × 10^−5^
		Q_e__(Cal)_ (mg/g)	118.5
		R^2^	0.96
		X^2^	1.05
N.C	Pseudo-first-order	K_1_ (1/min)	0.0056
		Q_e__Cal)_ (mg/g)	106.7
		R^2^	0.99
		X^2^	0.28
	Pseudo-second-order	k_2_ (mg/g min)	3.85 × 10^−5^
		Q_e (Cal)_ (mg/g)	134.37
		R^2^	0.96
		X^2^	0.97
S.C	Pseudo-first-order	K_1_ (1/min)	0.0053
		Q_e__(Cal)_ (mg/g)	129.34
		R^2^	0.99
		X^2^	0.037
	Pseudo-second-order	k_2_ (mg/g min)	3.12 × 10^−5^
		Q_e__(Cal)_ (mg/g)	161.99
		R^2^	0.99
		X^2^	0.25

#### 3.2.3 Classic equilibrium studies

##### 3.2.3.1 Effect of CFX concentration

**FIGURE 5:**
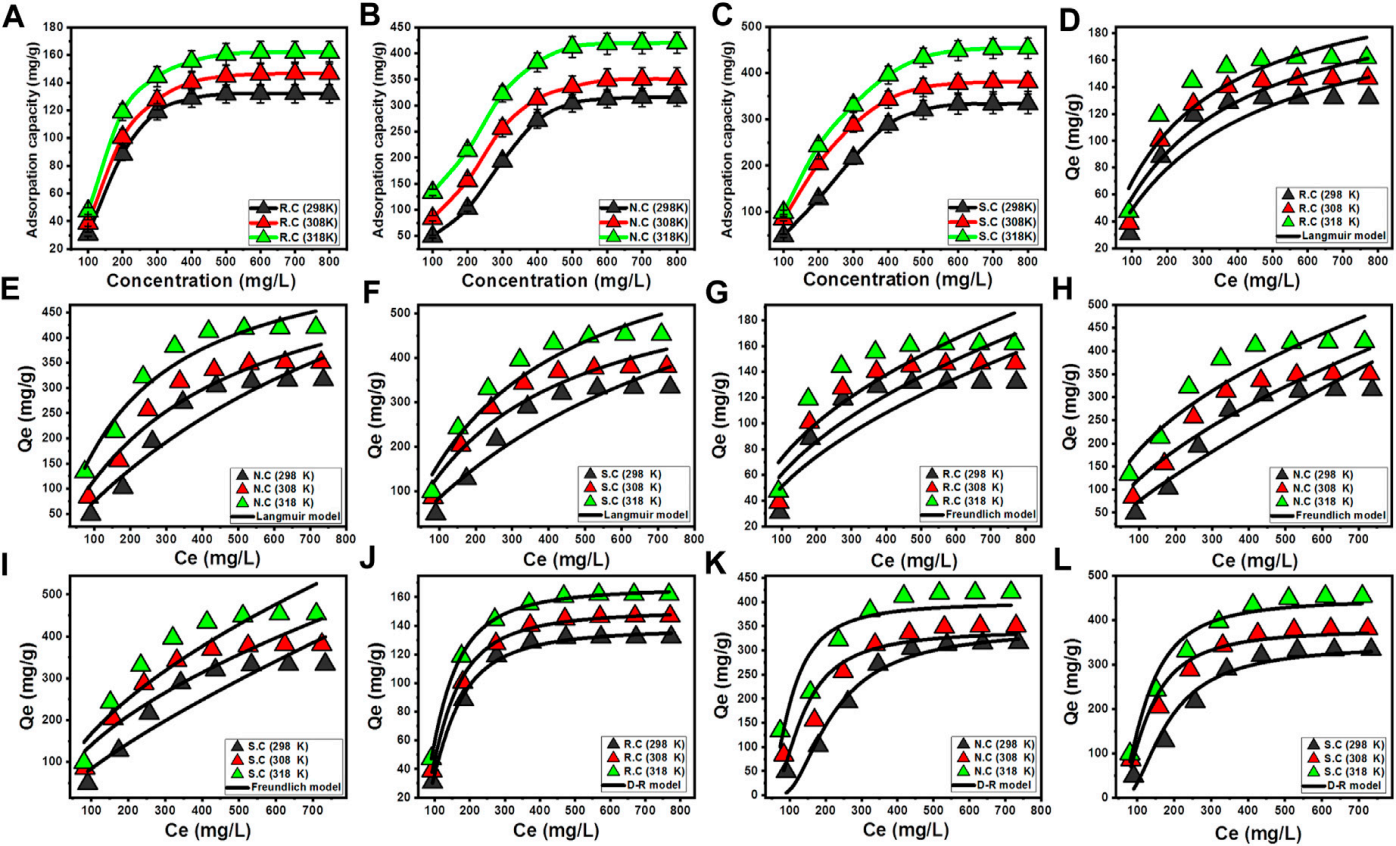
Influence of CFX concentrations on its adsorption capacities by R.C, N.C, and S.C **(A–C)**, fitting of the CFX adsorption results with the Langmuir model **(D–F)**, fitting of the CFX adsorption results with the Freundlich model **(G–I)**, and fitting of the CFX adsorption results with the Dubinin–Radushkevich (D–R) model **(J–L)**.

The experimental effect of the CFX starting concentration on the determined uptake capacities was assessed from 100 mg/L to 800 mg/L to denote the actual maximum capacities of R.C, N.C, and S.C, as well as the equilibrium properties of their adsorption systems. The essential experimental factors were considered at fixed values (0.2 g/L (coal dosage), pH 8 (adsorption pH), 1,080 min (adsorption duration), and 250 mL (volume)), while the adsorption temperature studied was within the range 298 K–318 K. The determined results experimentally validate the enhancement in the CFX uptake properties of R.C, N.C, and S.C by testing the adsorption of the antibiotic molecules at high starting concentrations (Figures 5A–C). This enhancement was documented widely in literature and illustrated based on the diffusion properties of the adsorbate as a function of its concentration. The driving forces and the diffusion behaviors of the CFX molecules increase strongly with the dissolution of the antibiotic at high concentrations. This, in turn, enhances the interaction and collision chances between the CFX ions and the effective receptors of R.C, N.C, and S.C (Ashraf et al., 2022). The experimental increment in the CFX uptake capacities continued until the investigated concentrations of 500 mg/L using R.C and up to 600 mg/L using both N.C and S.C. The determined CFX uptake capacities, after testing these concentrations, exhibit a neglected increase in the value or the same values, which signifies the previous concentrations as the equilibrium concentrations. During the identification of CFX equilibrium concentrations, all the free and effective adsorption receptors of R.C, as well as the modified samples of N.C and S.C, attain their full saturation or occupation with the antibiotic ions validating the experimental Q_max_ values of them.

The obtained Q_max_ values of CFX using R.C are 132 mg/g (298 K), 146.7 mg/g (308 K), and 161.8 mg/g (318 K) (Figure 5A). These values increased significantly by acid modification, with the determined values of N.C being 316 mg/g (298 K), 350.7 mg/g (308 K), and 420 mg/g (318 K) (Figure 5B), while the measured values using S.C are 333.8 mg/g (298 K), 380.6 mg/g (308 K), and 453.5 mg/g (318 K) (Figure 5C). These demonstrate the impacts of acid oxidation on enhancing the adsorption properties of raw coal either by enhancing the surface area or by the incorporation of numerous active oxygenated groups into the coal structure. The considerable enhancement in the determined CFX adsorption capacities by R.C, N.C, and S.C with increasing the adjusted adsorption temperature from 298 K to 318 K reflects the endothermic properties of coal adsorption systems.

##### 3.2.3.2 Classic isotherm models

The equilibrium properties of R.C, N.C, and S.C adsorption systems during the uptake were evaluated or modeled according to the reported hypothesis or assumptions of three widely used traditional isotherm models (Langmuir (Figures 5D–F), Freundlich (Figures 5G–I), and Dubinin–Radushkevich (D–R) (Figures 5J–L)). This was performed according to the non-linear fitting processes of the CRF removal results, with the representative equations of the previously mentioned models considering the obtained values of *R* ^2^ (determination coefficient) in addition to χ^2^ (Chi-squared) (Table 3). The adsorption of CFX by R.C, N.C, and S.C exhibits a better fitting with the equation and equilibrium assumption of Langmuir isotherm than the reported assumption and properties of Freundlich isotherm (Table 3). According to the equilibrium properties and suggestion of Langmuir isotherm, the CFX molecules were adsorbed homogeneously in monolayer forms by the present active centers on the surfaces of R.C, N.C, and S.C (Albukhari et al., 2021; Sayed et al., 2022). Moreover, the theoretically obtained RL parameter exhibits values less than 1, validating the favorable adsorption of CFX by R.C, N.C, and S.C. The theoretical Q_max_ of CFX using R.C, N.C, and S.C as estimated fitting parameters of the Langmuir model is 230.7 mg/g, 685.4 mg/g, and 854.2 mg/g, respectively, at the best-studied temperature (318 K) (Table 3).

**TABLE 3:** Mathematical parameters of the addressed classic isotherm models.

Classic isotherm models					
	**Models**	**Parameters**	**298 K**	**308 K**	**318 K**
R.C	Langmuir model	Q_max_ (mg/g)	210.3	220.3	230.7
		b(L/mg)	0.003	0.0035	0.0042
		R^2^	0.89	0.91	0.90
		X^2^	3.3	2.51	2.64
	Freundlich model	1/n	0.544	0.50	0.46
		k_F_ (mg/g)	4.15	5.97	8.81
		R^2^	0.82	0.83	0.81
		X^2^	5.5	4.7	5.04
	Dubinin–Radushkevich (D–R) model	β (mol^2^/KJ^2^) K	0.0545	0.048	0.042
		Q_m_ (mg/g)	137.85	150.5	166.5
		R^2^	0.99	0.99	0.99
		X^2^	0.07	0.04	0.008
		E (KJ/mol)	3.02	3.22	3.45
N.C	Langmuir model	Q_max_ (mg/g)	605	608.3	685.4
		b(L/mg)	0.0041	0.0023	9.19 × 10^−4^
		R^2^	0.96	0.95	0.93
		X^2^	2.27	3.5	4.81
	Freundlich model	1/n	0.80	0.60	0.47
		k_F_ (mg/g)	1.85	7.71	20.5
		R^2^	0.90	0.90	0.91
		X^2^	7.8	7.08	5.7
	Dubinin–Radushkevich (D-R) model	β (mol^2^/KJ^2^)	0.054	0.041	0.0304
		Q_m_ (mg/g)	341.7	352.2	399.3
		R^2^	0.91	0.93	0.89
		X^2^	5.4	3.8	6.6
		E (KJ/mol)	3.04	3.49	4.05
S.C	Langmuir model	Q_max_ (mg/g)	628.8	751.2	854.2
		b(L/mg)	0.0027	0.0027	0.001
		R^2^	0.94	0.94	0.93
		X^2^	4.86	5.3	6.2
	Freundlich model	1/n	0.78	0.57	0.58
		k_F_ (mg/g)	2.29	9.83	11.39
		R^2^	0.90	0.89	0.89
		X^2^	7.2	6.3	7.6
	Dubinin–Radushkevich (D-R) model	β (mol^2^/KJ^2^)	0.034	0.031	0.023
		Q_m_ (mg/g)	343.6	378.5	446.8
		R^2^	0.95	0.97	0.97
		X^2^	4.3	2.08	2.4
		E (KJ/mol)	3.83	4.01	4.66

The evaluation of the D–R equilibrium model strongly signifies the heterogeneity of the apparent energy during the adsorption reactions, either by the homogenous or heterogeneous surfaces of the used adsorbents (Dawodu et al., 2012). Moreover, the recognized fitting parameters of this model such as the Gaussian energy (E) validate the chemical and physical properties of the CFX adsorption processes by R.C, N.C, and S.C. The obtained Gaussian energy at values <8 kJ/mol, from 8–16 kJ/mol, and >16 kJ/mol suggested physisorption, weak chemisorption and ion exchange, and strong chemisorption mechanisms, respectively (Dawodu et al., 2012; Sayed et al., 2022). Therefore, the estimated E values of the CFX adsorption reactions by R.C (3.02–3.45 kJ/mol) as well as by N.C (3.04–4.05 kJ/mol) and S.C (3.83–4.66 kJ/mol) validate the antibiotic molecules by physisorption processes (Table 3).

#### 3.2.4 Advanced isotherm models

**FIGURE 6:**
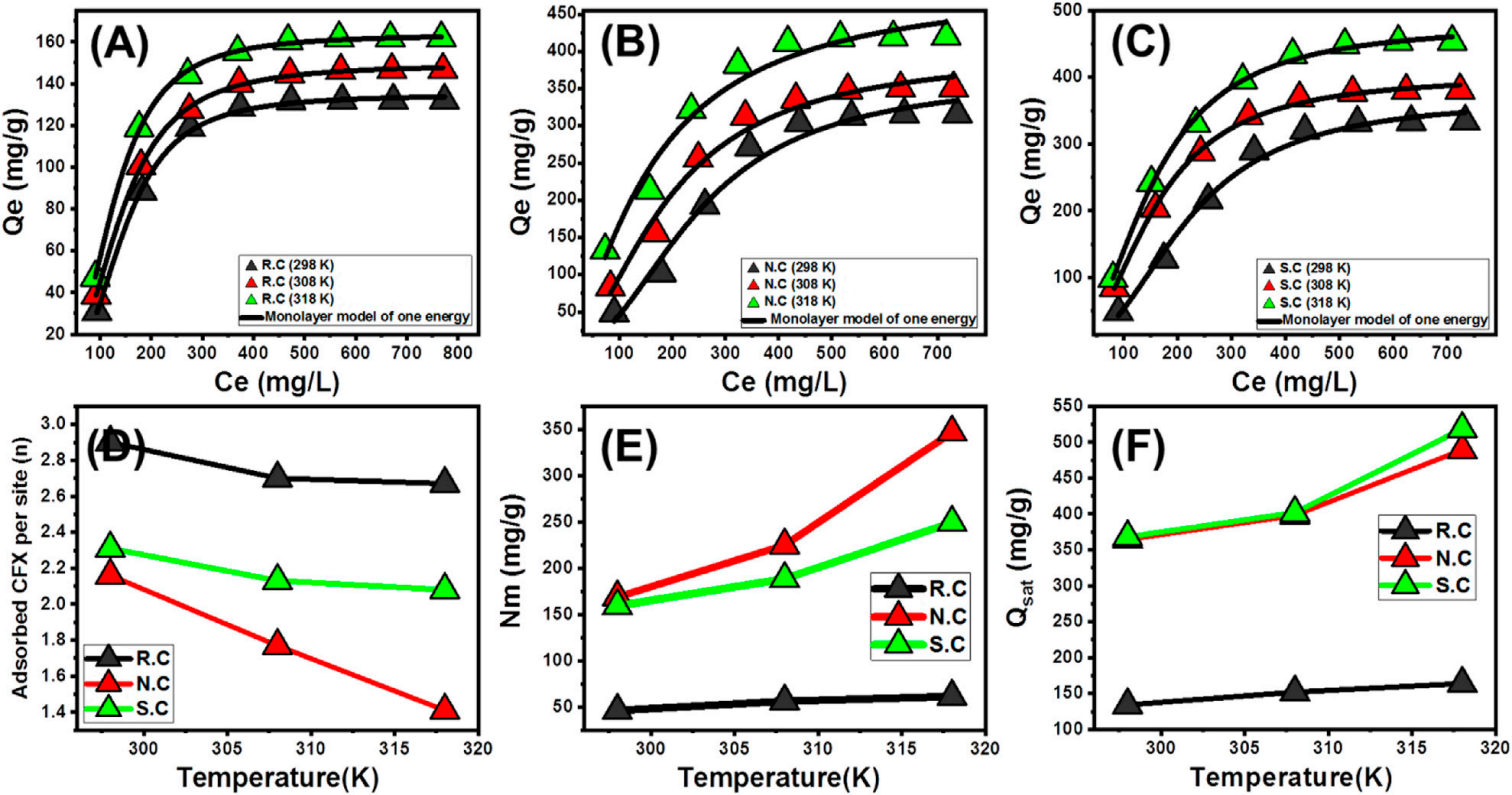
Fitting of the CFX uptake results with the advanced monolayer model of one energy site **(A–C)**, the change in the number of adsorbed CFX per site as a function of the temperature **(D)**, the change in the occupied adsorption sites density as a function of the temperature **(E)**, and the change in the adsorption capacities of the coal samples at their saturation states as a function of the temperature **(F)**.

In the later period, several advanced equilibrium models have been introduced considering the assumption and basics of the theory of statistical physics. These models are of deep significance in the adsorption mechanisms of CFX by R.C, N.C, and S.C in terms of the adsorbate/adsorbent interaction depending on the normally investigated mathematical steric parameters (occupied sites density (N_m_), number of adsorbed ions per site (n), and saturation adsorption capacity (Q_sat_)) and energetic parameters (internal energy (E_int_), adsorption energy (ΔE), free enthalpy (G), and entropy (Sa)) (Table 4). The fitting degrees with these models were identified based on the non-linear fitting results with the representative equations of these models using the Levenberg–Marquardt iterating algorithm as a function of multivariable non-linear regression. Based on the estimated *R* ^2^ and RMSE, the CFX adsorption processes by R.C as well as the oxidized coal samples (N.C and S.C) fit strongly with the monolayer model with one energy site (Figures 6A–C; Table 4).

**TABLE 4:** Estimated mathematical parameters for the fitting process with the monolayer model of one energy.

Steric and energetic parameters						
		**n**	**Nm (mg/g)**	**Q**_**sat**_ **(mg/g)**	**C1/2 (mg/L)**	**∆E (kJ/mol)**
R.C	298 K	2.9	46.32	134.33	143.42	3.49
	308 K	2.7	56.33	152.09	137.07	3.49
	318 K	2.67	61.44	164.04	125.61	3.37
N.C	298 K	2.16	168.73	364.54	245.41	4.82
	308 K	1.77	224.88	398.03	189.74	4.32
	318 K	1.41	346.98	489.24	159.09	4.00
S.C	298 K	2.31	159.27	367.9	216.29	4.51
	308 K	2.13	188.55	401.6	155.13	3.81
	318 K	2.08	249.29	518.5	152.57	3.89

##### 3.2.4.1 Steric parameters

###### 3.2.4.1.1 Number of adsorbed CFX molecules per site (n)

The estimated numbers of the CFX molecules (n_CFX_), which were adsorbed per each free and active uptake site of R.C, N.C, and S.C, significantly validate the orientation of the adsorbed CFX molecules in addition to the affected mechanism. The obtained values of n_CFX_ using R.C (*n* = 2.647–2.90), N.C (*n* = 1.41–2.16), and SS.C (*n* = 2.08–2.31) are higher than 1 (Figure 6D; Table 4). These n_CFX_ values demonstrate the orientation of the adsorbed CFX molecules in non-parallel and/or vertical form on the surfaces of R.C, N.C, and S.C by multi-molecular mechanisms (Sellaoui et al., 2016; Dhaouadi et al., 2020). Moreover, the active sites of R.C as well as the oxidized products of N.C and S.C can adsorb up to three molecules per site. There are remarkable decreases in the obtained n_CFX_ values in terms of the evaluated adsorption temperature from 298 K to 318 K using the three coal adsorbents (R.C, N.C, and S.C). This behavior validates a considerable decrease in the CFX aggregation properties during the uptake reactions using R.C, N.C, and S.C, as well as the high CFX adsorption rates before the aggregation of the molecules (Ali et al., 2021; Ashraf et al., 2022).

###### 3.2.4.1.2 Density of the adsorption sites (Nm)

The density of the occupied adsorption sites by CFX (Nm_(CFX)_) on the surfaces of R.C, N.C, and S.C significantly reflects the quantities of the effective active sites and validates the impact of the performed acid treatment processes either by HNO_3_ or by H_2_SO_4_ (Figure 6E; Table 4). The obtained Nm_(CFX)_ values of R.C are 46.32 mg/g (298 K), 56.33 mg/g (308 K), and 61.44 mg/g (318 K). These values enhanced significantly after the modification of coal with HNO_3_ (168.7 mg/g (298 K), 224.88 mg/g (308 K), and 364.9 mg/g (318 K)) and H_2_SO_4_ (159.2 mg/g (298 K), 188.55 mg/g (308 K), and 249.9 mg/g (318 K)) (Figure 6E; Table 4). This might be related to the activation effect of the used acids (HNO_3_ and H_2_SO_4_) as chemical activators on the surface of coal in addition to the significant incorporation of new forms of oxygenated, nitrogen-bearing, and sulfur-bearing chemical groups on its structure. The significant increase in the Nm _(CFX)_ values of R.C, N.C, and S.C in terms of the evaluated temperature from 298 K to 318 K was attributed to the observed decrease in the achieved n_CFX_ values. The increase in n_CFX_ values or the aggregation properties of CFX molecules resulted in a detectable decrease in the predicted numbers of the effective sites which were occupied during the captured antibiotic molecules (Ashraf et al., 2022). Moreover, other studies suggested a potential activation effect of the high-temperature conditions on the functional groups of R.C, N.C, and S.C (Dhaouadi et al., 2020). This might be caused by lowering the viscosity of the solutions and in turn increasing the mobility, diffusion rates, and collision chances between the CFX molecules and additional sites.

###### 3.2.4.1.3 The adsorption capacities at the saturation state of (Q_sat_)

The adsorption capacity of CFX at the saturation states of R.C, N.C, and S.C (Q_sat_) validates the most appropriate theoretical values of the maximum capacities of the studied products as adsorbents in comparison with the detected values based on the evaluated classic isotherm models. The Q_sat_ values of CFX estimated by R.C are 134.33 mg/g (298 K), 152.09 mg/g (308 K), and 164.04 mg/g (318 K). The estimated Q_sat_ values increased at a remarkable rate after the modification of coal with HNO_3_ (364.5 mg/g (298 K), 398.03 mg/g (308 K), and 489.24 mg/g (318 K)) and H_2_SO_4_ (367.9 mg/g (298 K), 401.6 mg/g (308 K), and 518.5 mg/g (318 K)). Considering the high dependence of the Q_sat_ values on the estimated n_CFX_ and Nm_(CFX)_ values, the remarkable enhancement in the estimated Q_sat_ of CFX by N.C and S.C can be attributed to the previously reported increase in the quantities of the effective sites by incorporating additional chemical groups as well as the reported enhancement in the surface area. However, the samples treated by HNO_3_ (N.C) exhibit higher Nm_(CFX)_ values than the samples treated by H_2_SO_4_ (S.C), which shows lower Q_sat_ values. This might be due to the higher surface area of S.C than of N.C in addition to the efficiency of the S.C adsorption sites to adsorb high numbers of CFX molecules than N.C.

These adsorption capacities were compared with those of other adsorbents in literature which demonstrate the higher efficiencies of the coal-acidified products as compared to several materials such as NH2-MIL-88B, magnetic mesoporous carbon, diamine-functionalized MCM-41, Fe_3_O_4_ nanoparticles, PSS-modified nano-alumina, and modified bentonite (Table 5). The presented results validate that the treated coal products, either by sulfuric acid or nitric acid, are low-cost and highly effective adsorbents for the antibiotic residuals from water and can be increased up to the realistic and commercial-scale applications.

**TABLE 5:** Comparison between the CFX adsorption capacities using the coal adsorbents and other materials in literature.

Adsorbent	Q _(max)_ (mg/g)	Reference
Modified bentonite	144.5	Antonelli et al. (2020)
PSS-modified nano-alumina	34.5	Nguyen et al. (2020)
Diamine-functionalized MCM-41	18.3	Lu et al. (2020)
Halloysite	21.7	Duan et al. (2018)
MMT clay	128	Gulen and Demircivi (2020)
**Fe**_3_O_4_ nanoparticles	24	Lin and Lee (2020)
Magnetic mesoporous carbon	98.28	Shi et al. (2013)
Moroccan oil shale	81.11	Chafyq et al. (2021)
Palygorskite–montmorillonite	107	Berhane et al. (2016)
Oat hulls	83	Movasaghi et al. (2019)
R.C	164.04	This study
N.C	489.24	This study
S.C	518.5	This study

##### 3.2.4.2 Energetic properties

###### 3.2.4.2.1 Adsorption energy

Adsorption energies (∆E) can interpret the adsorption mechanism (physical or chemical) that affects the adsorption of CFX by R.C, N.C, and S.C. For chemical adsorption, (ΔE > 80 kJ/mol), while for physical adsorption, (ΔE ≤ 40 kJ/mol). The physical adsorption may occur by coordination exchange (40 kJ/mol), hydrogen bonding (<30 kJ/mol), dipole forces (2–29 kJ/mol), van der Waals forces (4–10 kJ/mol), and hydrophobic bonds (5 kJ/mol) (Ali et al., 2021; Ashraf et al., 2022). The adsorption energy (∆E) can be calculated theoretically from Eq. 1 considering the values of the other variables such as the gas constant (R = 0.008314 kJ/mol.K), absolute temperature (T), solubility values of the adsorbates (S), and the concentrations of the adsorbates at the half-saturation states (Dhaouadi et al., 2020).

$$∆E=RT\;\ln\left(\frac{S}{C}\right).$$ (5)

The ΔE values of CFX calculated by R.C are 3.49 kJ/mol (298 K), 3.49 kJ/mol (308 K), and 3.37 kJ/mol (318 K). The estimated values of N.C are 4.82 kJ/mol (298 K), 4.32 kJ/mol (308 K), and 4 kJ/mol (318 K), while the obtained values during the adsorption of CFX by S.C are 4.51 kJ/mol (298 K), 3.81 kJ/mol (308 K), and 3.89 kJ/mol (318 K) (Table 5). These values indicate the dominant nature of the physical processes during the adsorption of CFX by R.C as well as by N.C and S.C (dipole bond forces, van der Waals forces, and hydrogen bonding).

###### 3.2.4.2.2 Thermodynamic functions

The entropy (Sa) properties of CFX adsorption systems by R.C, N.C, and S.C display the disorder and order properties of the surfaces of coal-based adsorbents at different concentrations of the adsorbates and different adsorption temperatures. The values of Sa can be calculated according to Eq. 6 using the previously obtained values of n, Nm_(CFX)_, and C_1/2_ (concentration at half saturation) (Sellaoui et al., 2020).

$$\frac{S_{a}}{K_{B}}=Nm\left\{\ln\left(1+{\left(\frac{C}{C_{1/2}}\right)}^{n}\right)-n{\left(\frac{C}{C_{1/2}}\right)}^{n}\;\frac{\ln\left(\frac{C}{C_{1/2}}\right)}{1+{\left(\frac{C}{C_{1/2}}\right)}^{n}}\right\}.$$ (6)

**FIGURE 7:**
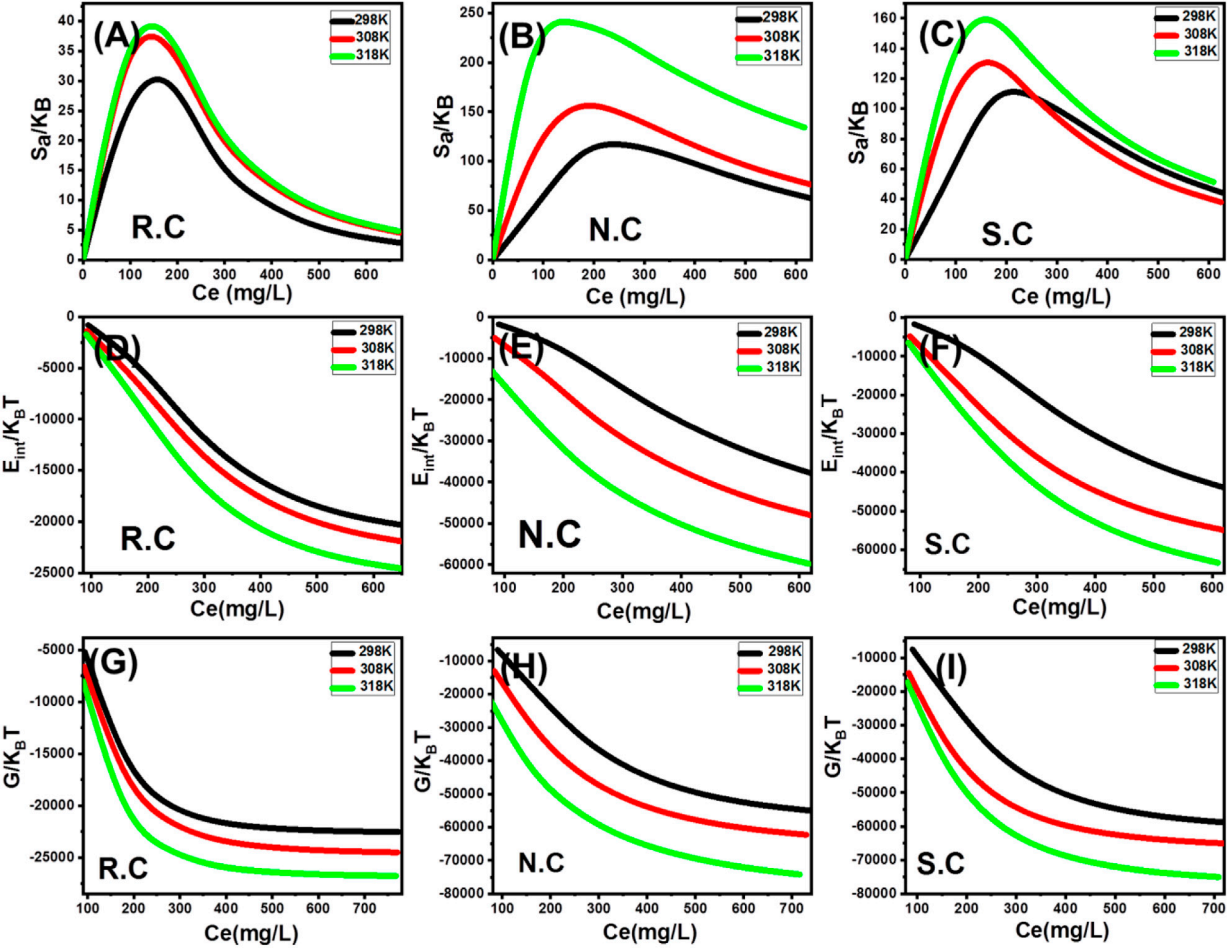
Change in the entropy values at different concentrations of CFX and at different temperatures **(A–C)**, change in the internal energy values at different concentrations of CFX and at different temperatures **(D–F)**, and change in the free enthalpy values at different concentrations of CFX and at different temperatures **(G–I)**.

The calculated values of Sa of the CFX adsorption reactions by R.C, N.C, and S.C decrease at considerable rates due to their high concentrations in the solutions (Figures 7A–C). Therefore, the disorder properties of the R.C, N.C, and S.C decrease significantly with the assessed high CFX concentrations. Also, this signifies the expected docking of CFX molecules on the free adsorption sites of R.C, N.C, and S.C at the low tested concentrations [59, 73]. The equilibrium CFX concentrations that are corresponding to the maxima values of Sa using R.C are 182.3 mg/L (298 K), 179.8 mg/L (308 K), and 176.2 mg/L (318 K). The estimated values using N.C are 261.3 mg/L (298 K), 248.7 mg/L (308 K), and 235.6 mg/L (318 K), while the recognized values using S.C are 256.7 mg/L (298 K), 159.3 mg/L (308 K), and 151.5 mg/L (318 K) (Figures 7A–C). The previously mentioned CFX concentrations are close to the estimated concentrations at the half saturation states of R.C, N.C, and S.C. Therefore, no additional CFX molecules can be docked on the active sites of coal-based adsorbents. Moreover, the steady declines in the Sa values reveal a remarkable decrease in both freedom degrees and diffusion properties of these ions, in addition to the strong depletion in the free adsorption sites (Sellaoui et al., 2016).

The internal energy (E_int_) and free enthalpy (G) properties of CFX adsorption processes by R.C, N.C, and S.C were evaluated based on the calculated values from Eqs.7, 8, respectively, using the previously obtained values of n, Nm_(CFX)_, and C_1/2_ (concentration at half saturation) in addition to the value of translation partition (Zv) (Sellaoui et al., 2020).

$$\frac{E_{\mathrm{int}}}{K_{B}T}=n\;N_{m}\left[\left(\frac{{\left(\frac{C}{C_{1/2}}\right)}^{n}\;\ln\left(\frac{C}{Z_{v}}\right)}{1+{\left(\frac{C}{C_{1/2}}\right)}^{n}}\right)-\left(\frac{n\ln\left(\frac{C}{C_{1/2}}\right)\;{\left(\frac{C}{C_{1/2}}\right)}^{n}}{1+{\left(\frac{C}{C_{1/2}}\right)}^{n}}\right)\right],$$ (7)

$$\frac{G}{K_{B}T}=n\;N_{m}\frac{\ln\left(\frac{C}{Z_{v}}\right)}{1+{\left(\frac{C_{1/2}}{C}\right)}^{n}}.$$ (8)

The recognized E_int_ values of CFX adsorption reactions by R.C, N.C, and S.C exhibit negative signs and increase regularly with the temperature ranging from 298 K to 318 K. Based on these results and behaviors, the R.C, N.C, and S.C adsorption systems of CFX show spontaneous and endothermic behaviors (Figures 7D–F). The recognized free enthalpy values (G) demonstrate the same behaviors and findings (Figures 7G–I). The negative signs of G values as well as the observed increase in these values with the adsorption temperature confirm the spontaneous and endothermic properties of the studied adsorption systems of R.C, N.C, and S.C in addition to the considerable increase in the feasibility properties of these reactions (Figures 7G–I).

#### 3.2.5 Recyclability

**FIGURE 8:**
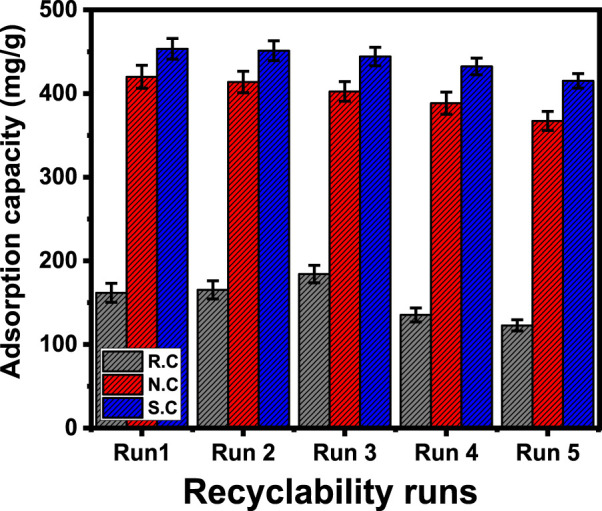
Recyclability properties of raw coal and the modified products during the adsorption of CFX.

The reusability properties of the studied R.C, N.C, and S.C as adsorbents of CFX were a vital factor during the realistic and industrial-scale assessment of the products. The spent fractions of the coal-based adsorbents were washed extensively with distilled water for 10 min, which was repeated three times. Afterward, the washed products were dried for 10 h at 65°C and then reused again in the subsequent CFX adsorption cycle. The reusability tests were completed at fixed experimental conditions (volume: 250 mL; dosage: 0.2 g/L; contact time: 1,080 min; pH 8; concentration 800 mg/L; and temperature: 318.13 K). The reusability tests validate significant stabilities of R.C, N.C, and S.C as adsorbents of CFX residuals from water. The determined CX uptake capacities during the recyclability of R.C are 161.8, 156.3, 148.2, 135.3, and 122.8 mg/g for runs 1–5, respectively (Figure 8). For N.C, the obtained CFX uptake capacities are 420, 413.7, 402.5, 388.5, and 367.2 mg/g for runs 1–5, respectively (Figure 8). For the recyclability of S.C, the measured CFX uptake capacities are 453.5, 451.2, 444.3, 432.4, and 415.2 mg/g for runs 1–5, respectively (Figure 8). The decrease in the CFX adsorption properties of R.C, N.C, and S.C was observed with the regular repetition of the reusability runs. This behavior was assigned to the expected loss in the weights of the adsorbents during the performed washing and regeneration steps in addition to the expected increase in the quantities of the formed chemical complexes with CFX chemical structure.

#### 3.2.6 The CFX adsorption mechanism

**FIGURE 9:**
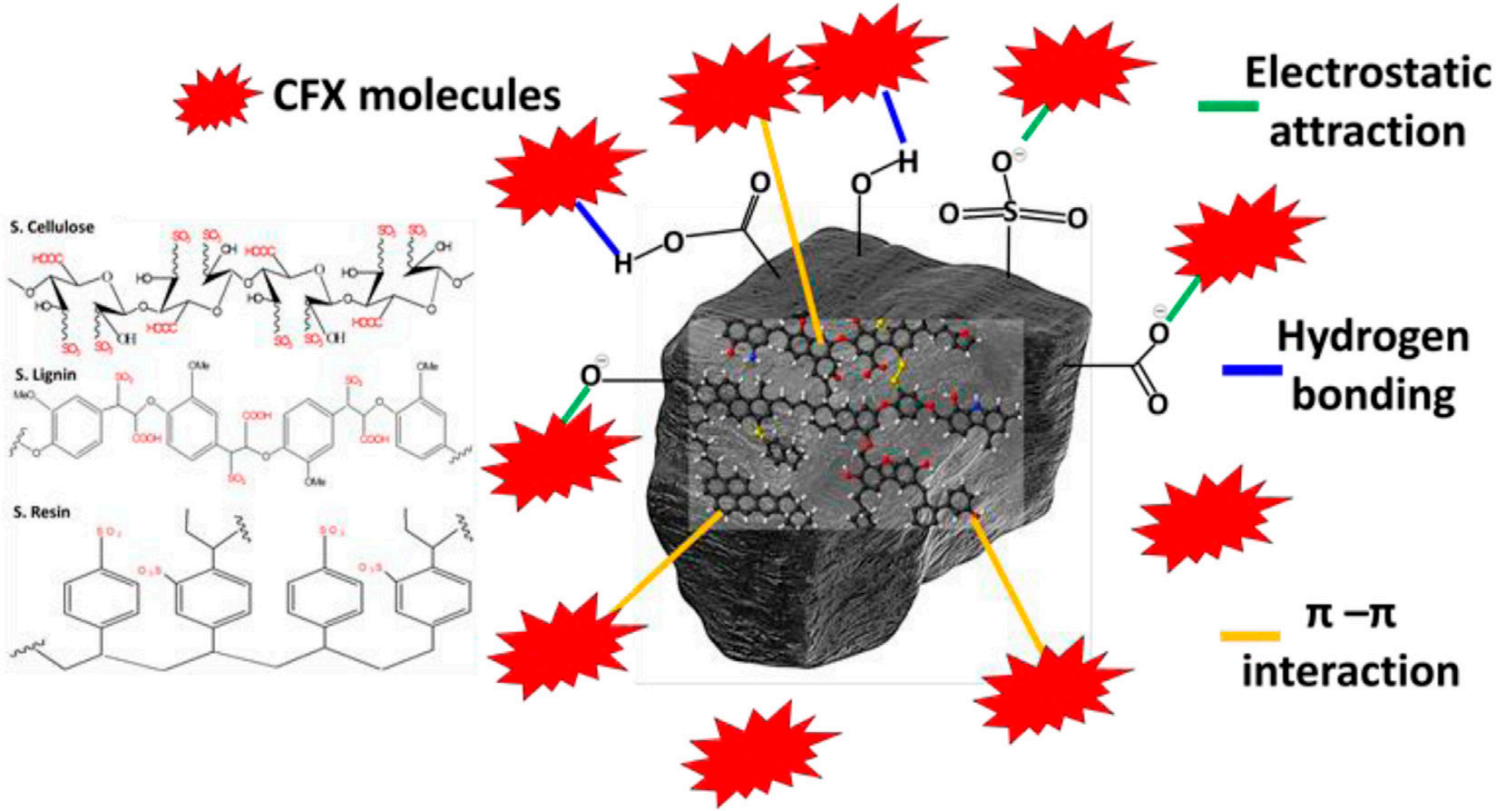
Schematic for the adsorption mechanism of CFX molecules by the modified coal products.

The modified coal samples either by nitric acid (N.C) or sulfuric acid (S.C) contain several species of active oxygenated chemical groups such as–COOH, –OH^−^, and–SO_3_H in addition to other structural chemical groups and the incorporated nitrogen and sulfur chemical groups (Figure 9). Considering the dominant chemical groups, theoretical mechanistic findings according to the equilibrium studies, and the other literature, the adsorption of CFX molecules on the surface of both N.C and S.C can occur by three essential processes (Figure 9). The first mechanism involved the strong electrostatic attractions of the CFX basic ions by the active and negatively charged oxygenated chemical groups as well as the entrapped sulfur- and nitrogen- bearing chemical groups. The second suggested mechanism involved formation of hydrogen bonds between the structural nitrogen atoms within the chemical structure of CFX and the free hydrogen ion on the surface of the oxidized coal particles. The third process that might affect the adsorption of CFX is the π–π interaction, which occurred between the π-electrons on the surface of oxygenated coal and the structural CFX aromatic rings. This interaction involved significant complex dispersion and dipole-induced dipole attraction processes (Figure 9).

## 4 Conclusion

H_2_SO_4_- and HNO_3_-modified coal products were prepared as enhanced and low-cost adsorbents of ciprofloxacin residuals. The modified coal samples exhibit enhanced CFX adsorption capacities (N.C (489.2 mg/g) and S.C (518.5 mg/g)) as compared to raw coal (164.08 mg/g). The adsorption systems, in terms of adsorbate/adsorbent interactions, were illustrated based on the steric and energetic properties. Based on the CFX-occupied active site’s density (Nm_[_), the modified samples, especially by nitric acid show significant increment in the active sites [N.C (364.9 mg/g) and S.C (249.9 mg/g)] as compared to R.C (61.44 mg/g). The observable higher capacities of the actives sites on the surface of S.C to adsorb more CFX molecules (*n* = 2.08–2.31) than N.C (*n* = 1.41–2.16) illustrate the determined higher adsorption capacity. The values of the adsorption (˂40 kJ/mol) and Gaussian (˂8 kJ/mol) energies demonstrate the dominant effect of the physisorption mechanism, and the thermodynamic function validates the spontaneous and endothermic properties of these processes.

## Data Availability

The raw data supporting the conclusion of this article will be made available by the authors, without undue reservation.
