# Supplementary material for: Insight into the effects of H2SO4 and HNO3 acidification processes on the properties of coal as an enhanced adsorbent for ciprofloxacin residuals: Steric and energetic studies
Source: Front Chem. 2023 Mar 27;11:1130682. doi: 10.3389/fchem.2023.1130682 (PMC10083360; doi:10.3389/fchem.2023.1130682)
Supplement: Supplementary file 1 [file Table1.DOCX]

**Insight into the effect of the H_2_SO_4_ and HNO_3_ acidification processes on the properties of coal as an enhanced adsorbent for Ciprofloxacin residuals: steric and energetic studies**

**Ibrahim G. Al-Labadi^1^, Marwa H. Shemy^2,3^,** **Alaa Y. Ghidan^4^, Ahmed A. Allam^5^,** **Horvath M. Kalman^1^, Jamaan S. Ajarem^6^, Jianmin Luo^7^, Chuanyi Wang^8^, Mostafa R. Abukhadra^2,9^٭**

^1^Department of Environmental Analysis and Technologies, Institute of Environmental Sciences, Hungarian University of Agriculture and Life Sciences, Páter Károly u. 1, 2100 Gödöllő, Hungary

^2^Materials Technologies and their Applications Lab, Geology Department, Faculty of Science, Beni-Suef University, Beni-Suef City, Egypt.

^3^Chemistry Department, Faculty of Science, Beni-Suef University, Beni-Suef City, Egypt

^4^Department of Biological Sciences, Faculty of Sciences, The University of Jordan, Amman, 11942

^5^Zoology Department, Faculty of Science, Beni-Suef University, Beni-Suef, Egypt

^6^Zoology Department, College of Science, King Saud University, Riyadh, Saudi Arabia

^7^School of Chemistry and Civil Engineering, Shaoguan University, Shaoguan 512005, P. R. China

^8^School of Environmental Science and Engineering, Shaanxi University of Science and Technology, Xi’an 710021, China

^9^Geology Department, Faculty of Science, Beni-Suef University, Beni-Suef, 65211, Egypt

Corresponding author٭.Tel: +2001288447189. E-mail: [Abukhadra89@Science.bsu.edu.eg](mailto:Abukhadra89@Science.bsu.edu.eg)

**Table.S1.** Nonlinear equations of kinetic, classic isotherm, and advanced isotherm models

| Kinetic models | | |
| --- | --- | --- |
| Model | **Equation** | **Parameters** |
| Pseudo-first-order | $Q_{t}=Q_{e} (1-e^{{-k}_{1}.t})$ | Q_t_ (mg/g) is the adsorbed ions at time (t), and K_1_ is the rate constant of the first-order adsorption (1/min) |
| Pseudo-second-order | $Q_{t}=\frac{Q_{e}^{2}k_{2}t}{1+Q_{e}k_{2}t}$ | Qe is the quantity of adsorbed ions after equilibration (mg/g), and K_2_ is the model rate constant (g/mg min). |
| Classic Isotherm models | | |
| Model | **Equation** | **Parameters** |
| Langmuir | $Q_{e}=\frac{Q_{max} bC_{e}}{(1+bC_{e})}$ | *C_e_* is the rest ions concentrations (mg/L), *Q_max_* is the theoritical maximum adsorption capacity (mg/g), and *b* is the Langmuir constant (L/mg) |
| Freundlich | $Q_{e}=K_{f}C_{e}^{1/n}$ | K_F_ (mg/g) is the constant of Freundlich model related to the adsorption capacity and n is the constant of Freundlich model related to the adsorption intensities |
| Dubinin–Radushkevich | $Q_{e}=Q_{m}e^{-\betaɛ^{2}}$ | β (mol^2^/KJ^2^) is the D-R constant, ɛ (KJ^2^/mol^2^) is the polanyil potential, and Q_m_ is the adsorption capacity (mg/g) |
| Advanced isotherm models | | |
| Model | **Equation** | **Parameters** |
| Monolayer model with one energy site (Model 1) | $Q=nN_{o} =\frac{nN_{M}}{1+{(\frac{C1/2}{C})}^{n}}=\frac{Q_{o}}{1+{(\frac{C1/2}{C})}^{n}}$ | Q is the adsorbed quantities in mg/g  n is the number of adsorbed ion per site  Nm is the density of the effective receptor sites (mg/g)  Q_o_ is the adsorption capacity at the saturation state in mg/g  C1/2 is the concentration of the ions at half saturation stage in mg/L  C1 and C2 are the concentrations of the ions at the half saturation stage for the first active sites and the second active sites, respectively  n1 and n2 are the adsorbed ions per site for the first active sites and the second active sites, respectively |
| Monolayer model with two energy sites (Model 2) | $Q=\frac{n_{1}N_{1M}}{1+{(\frac{C_{1}}{C})}^{n_{1}}}+\frac{n_{2}N_{2M}}{1+{(\frac{C_{2}}{C})}^{n_{2}}}$ |  |
| Double layer model with one energy site (Model 3) | $Q=Q_{o}\frac{({\frac{C}{C1/2})}^{n}+2({\frac{C}{C1/2})}^{2n}}{1+({\frac{C}{C1/2})}^{n}+({\frac{C}{C1/2})}^{2n}}$ |  |
| Double layer model with two energy sites (Model 3) | $Q=Q_{o}\frac{({\frac{C}{C1})}^{n}+2({\frac{C}{C2})}^{2n}}{1+({\frac{C}{C1})}^{n}+({\frac{C}{C2})}^{2n}}$ |  |
